# A Comparison of Low-Temperature Deformation Behavior and Fracture in Low-Carbon Steel Specimens Obtained by Electron Beam Additive Manufacturing and Conventional Casting and Normalization

**DOI:** 10.3390/ma16010446

**Published:** 2023-01-03

**Authors:** Elena Astafurova, Kseniya Reunova, Evgenii Melnikov, Marina Panchenko, Sergey Astafurov, Andrey Luchin, Elena Zagibalova, Evgenii Kolubaev

**Affiliations:** Institute of Strength Physics and Materials Science, Siberian Branch of Russian Academy of Sciences, 634055 Tomsk, Russia

**Keywords:** low-carbon steel, microstructure, mechanical properties, fracture, electron beam additive manufacturing

## Abstract

In the present work, the microstructure, phase composition, and temperature dependence of the mechanical properties and fracture micromechanisms of low-carbon steel produced by conventional casting and electron beam additive manufacturing have been studied. Regardless of the manufacturing method, the phase composition of steel consists of ferrite with an insignificant fraction of carbides (pearlite grains in both types of steel and single coarse precipitates in the additively fabricated one). It was shown that the studied steels are characterized by a strong temperature dependence on yield strength and ultimate tensile strength. At T = 77 K, both types of steel are characterized by high strength properties, which decrease with increasing test temperatures up to 300 K. In addition, all deformation curves are characterized by the presence of a yield drop and yield plateau over the entire temperature range under study (77 K–300 K). A decrease in test temperature from 300 K to 77 K leads to a change in the fracture micromechanism of the steels from a dimple fracture to a cleavage one. Despite the similar deformation behavior and strength properties, the additively fabricated steel possesses lower elongation to failure at 77 K due to an insignificant fraction of coarse precipitates, which assists the nucleation of brittle cracks.

## 1. Introduction

Additive manufacturing (AM) is a relatively new technology that is based on the layer-by-layer deposition of a material on a substrate according to a three-dimensional computer model [1,2,3]. AM is a universal production method, as it gives the opportunity to form complex-shaped products of different scales using various raw materials, such as metals, alloys, composites, ceramics, etc. [1,4]. Nowadays, modern AM technologies are widely used in the medical, automotive, aerospace, and other industries [5].

There are several types of AM methods, which depend on the tasks to be solved. Selective laser melting [6,7] based on a powder bed fusion process and laser direct energy deposition [8], which allows a synchronous feeding of raw materials and their melting by a laser beam, are suitable for the precise fabrication of small components. Wire and arc additive manufacturing, which are superior techniques for prototyping large-scale metal objects, allow for achieving better performance and high process rates [9,10]. Electron beam additive manufacturing (EBAM) is among the most prospective methods of AM [11,12]. The high processing rate, the possibility to use several raw materials in one processing route, the high-vacuum protective atmosphere, and the possibility to obtain large parts are characteristics of the EBAM process [1,11].

Low-carbon steels are widely used materials in mechanical engineering due to their moderate mechanical properties and good weldability [13]. They are indispensable construction materials for bridges, ships, pressure vessels, etc. The conventional industrial production of low-carbon steel components is an energy-consuming and labor-intensive process which includes casting, thermal-mechanical treatments, extractive metal processing, and welding. Therefore, manufacturers are keen to find and develop more economical and efficient methods for production of the final products and billets from this type of construction material. AM could be an excellent way to produce parts made from low-carbon steels. Currently, there are just a few works devoted to the study of the properties of EBAM-fabricated low-carbon steel [14,15]. EBAM has been used to obtain billets from low-carbon steel with a constant phase composition (ferrite with carbides), and the mechanical properties of the additively manufactured specimens are slightly lower than those in the conventionally produced specimens with a ferritic–pearlitic structure [14]. In [15], the authors showed that the microstructure of the EBAM-produced low-carbon steel is heterogeneous and consisted of grains of ferrite and pearlite. In addition, it was shown that low-carbon steel by the EBAM process is characterized by relatively low strength and high ductility. Thus, the investigation of a microstructure and the mechanical properties of low-carbon steels produced using EBAM technology is relevant.

The aim of this paper is to study the temperature dependence of the deformation behavior, the fracture mechanisms, and the mechanical properties of low-carbon steel obtained by the EBAM method and to compare these parameters with data for conventionally fabricated low-carbon steel of a similar composition (normalized with a ferritic–pearlitic structure).

## 2. Materials and Methods

**Figure 1:**
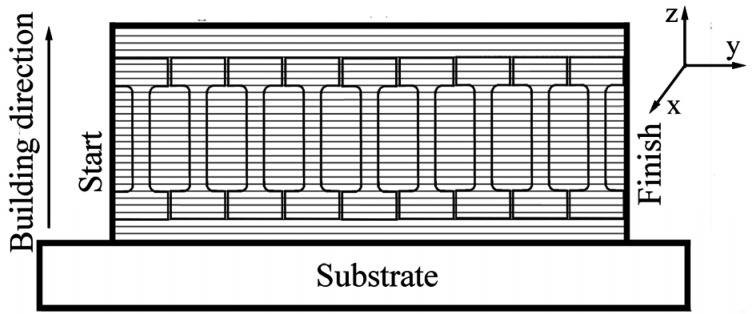
A scheme of specimen orientation relative to the building direction of the billet.

Three billets (walls) with linear dimensions of 110 × 30 × 5 mm^3^ were produced using EBAM with a sequential layer-by-layer deposition of 30 parallel layers (Figure 1).

A welding wire Fe-(1.8–2.1)Mn-(0.7–0.95)Si-(0.05–0.11)C (wt.%) with a diameter of 1.2 mm was used as a raw material for EBAM. The AM process was carried out in vacuum (P = 1 × 10^−3^ Pa) with the following technological parameters: beam current I = 44–50 mA, accelerating voltage U = 30 kV, wire-feed rate V_w_ = 5.8 mm/s, ellipse scan 4 × 4 mm, and scanning frequency—1 kHz. Austenitic stainless steel plate was used as a substrate material; it was not cooled during the EBAM process. The EBAM-fabricated billet (and specimens) was not subjected to any thermal treatment and was studied in the as-built condition.

To compare the mechanical properties of the additively produced low-carbon steel with that obtained by the conventional cast production, specimens of hot-rolled Fe-(1.3–1.7)Mn-(0.5–0.8)Si- < 0.12 C (wt.%) steel were used. These specimens were normalized for 30 min at 980 °C followed by air cooling to produce a ferritic–pearlitic structure (further called the cast specimens). The comparison of the additively manufactured steel specimens with those obtained by the conventional production technique is important for substantiation of the possibility of using new production technologies (electron beam additive manufacturing) in the production of parts made from industrially important materials.

The compositions of the wire for the EBAM process and cast specimens were measured using spectrometer HG-Profiler 2 (Horiba). The differences in the elemental compositions of the wire and the cast specimens are insignificant in affecting the mechanical properties and microstructure of the steel.

**Figure 2:**
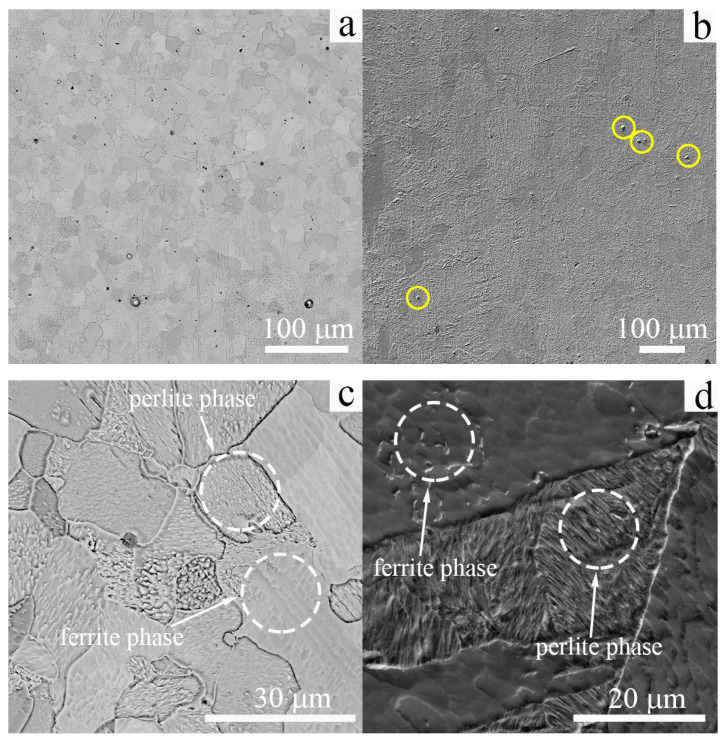
SEM images of the microstructure of the low-carbon steel: (**a**,**c**) cast and (**b**,**d**) EBAM. Yellow circles in (**b**) show the coarse carbide particles.

Flat, proportional, dumbbell-shaped specimens with gauge sections of 12 × 2.7 × 1.3 mm^3^ (length × height × thickness) were cut from the walls (further called EBAM-fabricated specimens). All specimens were oriented along the building direction of the billet (Figure 2). All specimens were mechanically ground using abrasive papers and electrochemically polished in a 50 g CrO_3_ + 200 g H_3_PO_4_ solution.

Tensile mechanical tests were carried out at 77, 183, 223, 273, and 300 K and at initial strain rate of 5 × 10^−4^ s^−1^. For the mechanical tests, an electromechanical machine (Instron 1185) equipped with a cryogenic chamber was used. During the test, the testing system (specimen and specimens’ holder) was set in a chamber filled with liquified nitrogen (for the test temperature 77 K) or ethanol cooled with the liquified nitrogen (for intermediate temperature). The test temperature was kept constant during the active tension (temperature change did not exceed 2 K). To control the temperature during active tension, a thermocouple was used; it was fixed near the working part of the specimen and was not removed from the chamber during the test.

A scanning electron microscope (SEM, Thermo Fisher Scientific Apreo S LoVac (Waltham, MA, USA) equipped with an electron backscattered diffraction (EBSD) unit was used for investigation of the grain structure in the materials, and fracture surfaces of the specimens tensile tested to failure. A diffractometer DRON 7 (Bourevestnik) was used for X-ray diffraction (XRD) analysis of the specimens (in a θ–2θ Bragg-Brentano geometry, Co-K_α_ radiation). To study the phase composition of the steel after the EBAM process by XRD methods, the specimens (10 × 5 × 1 mm^3^) were cut, and the plane surfaces of these specimens were lain in the planes (xy), (xz), and (zy) (Figure 1).

## 3. Results and Discussion

### 3.1. Microstructure and Phase Composition of Low-Carbon Steel

Figure 2 demonstrates SEM images of the microstructures in the steel specimens in the cast state and after the EBAM process. A ferritic phase is the dominating one, and the microstructure of the low-carbon steel is homogeneous in both types of specimens.

In the cast state, the average grain size of the ferritic phase is 14 ± 6 μm, and it is 25 ± 12 μm for the specimens obtained by the EBAM technique (Figure 2a,b). Due to the differences in production methods, the grain size of the cast specimens is two-times lower as that in the EBAM fabricated one. This difference arises due to the hot rolling of the cast specimens, which is typically used in conventional steel production for the homogenization of the ingots. This treatment assists grain refinement in the cast steel relative to the EBAM-fabricated specimens, in which the grain structure forms during the crystallization of the melting pool and multiple cooling/heating cycles of the billet (recrystallization in a solid state).

A ferritic–pearlitic microstructure is typical of conventionally fabricated low-carbon steel subjected to normalization treatment (Figure 2c). In the additively manufactured specimens, some grains are characterized by a specific pearlitic relief as well (Figure 2d), and large inclusions of the carbide particles are also seen in the SEM images (Figure 2b). Some of these carbide particles are highlighted by the yellow circles in Figure 2b. In the SEM images shown in Figure 2d, the regions of the pearlitic phase have a needle-like morphology with interlayers several micrometers thick. A similar microstructure of the EBAM-fabricated low-carbon steel was reported in [14].

**Figure 3:**
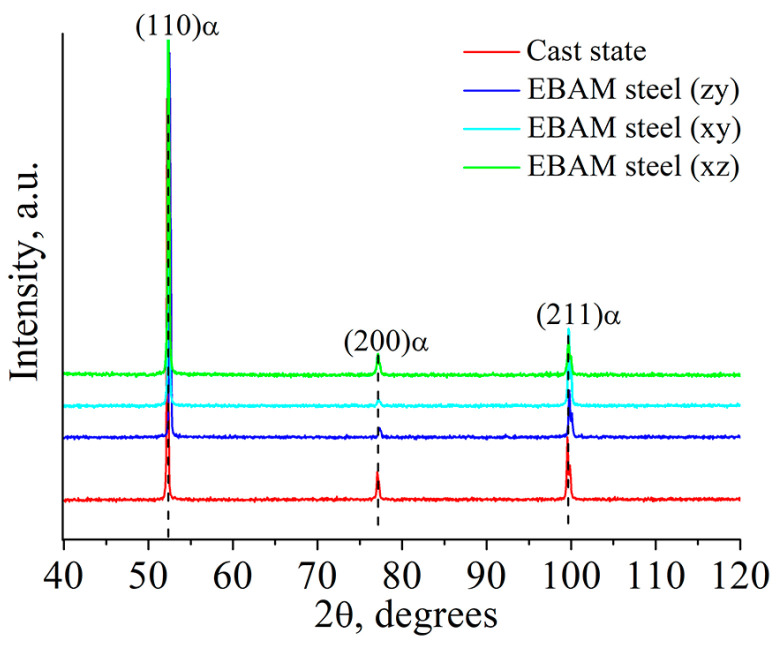
XRD patterns obtained for low-carbon steel in the cast state and after the EBAM process.

According to the XRD data presented in Figure 3, ferrite with a bcc crystal lattice is the basic phase of the low-carbon steel both in the cast state and after the EBAM process. A crystal lattice parameter of the ferritic phase in the cast specimens is 2.8675 ± 0.0003 Å. Specimens of the additively manufactured steel have close crystal lattice parameters—2.8683 ± 0.0004 Å. The absence of XRD reflections corresponding to other phases indicates that the volume content of the carbides (in coarse particles and pearlite) is less than 5 vol.%. In addition, no differences in the crystal lattice parameters, relative intensities of the XRD peaks, or phase composition were indicated for the EBAM-fabricated specimens, which were cut in different directions. This testifies to the isotropy of the grain structure of low-carbon steel produced by EBAM, which distinguishes it from the highly anisotropic austenitic stainless steels fabricated by AM [16].

Thus, it can be concluded that low-carbon steel obtained by the EBAM method has an equiaxed misoriented structure, and its phase composition (and grain size) is similar to that of conventionally produced low-carbon steel with a ferritic–pearlitic structure.

### 3.2. Temperature Dependence of Deformation Behavior and Mechanical Properties of Low-Carbon Steel

**Figure 4:**
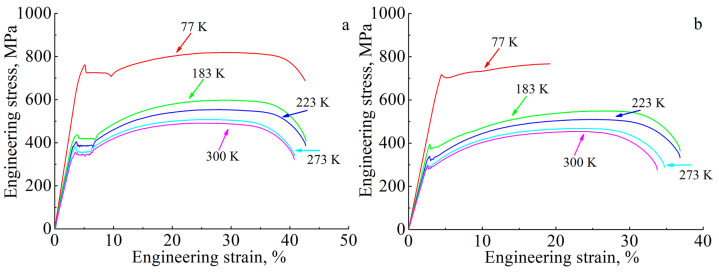
Tensile “engineering stress vs. engineering strain” diagrams for the low-carbon steel: (**a**) steel in the cast state and (**b**) steel after the EBAM process.

The “engineering stress vs. engineering strain” diagrams for the low-carbon steel in the cast state and after the EBAM process is shown in Figure 4 for the five test temperatures. The diagrams have the form and stages of the plastic flow typical for normalized low-carbon steels: a yield drop, a yield plateau, and subsequent parabolic hardening. The yield drop is a sharp transition between elastic and plastic deformation followed by a horizontal section of the stress-strain diagram (the yield plateau). The appearance of the yield phenomena is typical tensile behavior of many materials and is associated with the “unpinning” of dislocations from the atmospheres of carbon and manganese atoms and the formation of Chernov–Lüders bands. During the deformation stage corresponding to the yield plateau, a front of the localized deformation (or several fronts) propagates in the specimen until all its volume is involved in the plastic deformation. Then, the stage of parabolic hardening starts and lasts until an ultimate tensile strength is reached, which corresponds to the maximum of the engineering stress. Then, the prefracture stage (with the neck formation) begins, and the deforming stresses decrease with strain due to the neck formation [13,17].

**Figure 5:**
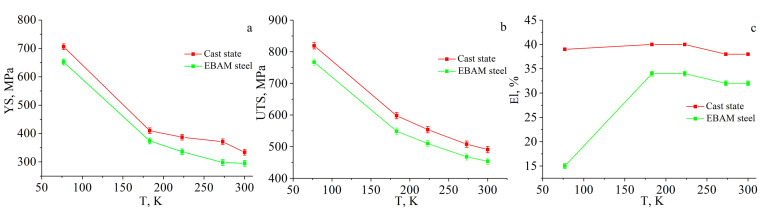
Temperature dependences of the mechanical properties of the low-carbon steel in the cast state and after the EBAM process: (**a**) the yield strength (YS), (**b**) the ultimate tensile strength (UTS), and (**c**) the elongation to failure (El).

Regardless of the manufacturing method, a strong temperature dependence on the mechanical properties is typical for low-carbon steel (Figure 4). As can be seen from Figure 4a and Table 1, the form of the diagrams, stages of plastic flow, and plasticity are practically independent of the test temperature for the cast specimens. The maximum values of yield strength and ultimate tensile strength are observed at T = 77 K, and these characteristics decrease with an increase in test temperature (Figure 5a,b). In addition, the yield drop and yield plateau are observed on the tensile diagrams for all test temperatures. The decrease in the test temperature down to T = 77 K contributes to a growth of the yield drop and an increase in the length of the yield plateau (Figure 4a, Table 1). 

Additively manufactured low-carbon steel exhibits similar mechanical behavior. As in the case of the cast steel, the EBAM-fabricated specimens have the highest values of strength properties at T = 77 K; however, the elongation-to-fracture at this test temperature is reduced relative to the higher test temperatures (Figure 4b and Figure 5 and Table 1). An increase in test temperature leads to a decrease in the yield strength and the ultimate tensile strength. It also causes a two-fold increase in elongation-to-failure in the temperature range 77–183 K with a further reach to some near-stationary value of this parameter at T > 183 K (Figure 4b and Figure 5b and Table 1). 

Analysis of data in Table 1 and Figure 5 shows that the EBAM-fabricated specimens of low-carbon steel demonstrate lower values for the strength characteristics and elongation in comparison with the cast steel within the whole temperature interval (on average, no more than 15% for each parameter excluding the elongation value at a temperature of 77 K). A decrease in the yield strength could be associated with the difference in the grain sizes of the EBAM-fabricated and cast specimens. According to the Hall–Petch relationship [18,19], the YS of a polycrystalline material depends on the grain size *d* according to the following law—YS~1/*d*^1/2^. The difference in grain sizes of the specimens is not so high (14 μm vs. 25 μm) but is sufficient to cause about a 50 MPa [13] decrease of the YS in the EBAM-fabricated material relative to the cast one. The other hardening mechanisms can be neglected. Namely, the close values of the lattice constants for the cast and the EBAM-fabricated steels (*a* = 2.8683 Å) show that a solid-solution hardening is similar for them. Particle strengthening also has a minor effect because of the low volume fraction and the big size of the carbides in the ferritic grains.

The coarse carbides in the additively manufactured low-carbon steel could decrease the ductility of the specimens under loading because such inclusions act as strong stress concentrators in the ferritic matrix [20,21]. With the increase of stresses applied during the tensile deformation, the stress fields of the elevated stresses are formed around such particles, which promote a local plastic flow near the carbides and cause a premature failure of the specimens (especially in the low-temperature deformation regime, where applied stresses are the highest).

In general, the stages of plastic flow in low-carbon steel produced by EBAM remain the same as in cast steel. However, at the temperature of 77 K, the value of the yield drop for additively manufactured steel is smaller than that for the cast one, and the length of the yield plateau is noticeably shorter over the entire temperature range (Table 1). Such changing of parameters of the yield phenomenon are apparently associated with the fact that most carbon atoms are bounded into carbides in additively manufactured low-carbon steel, and the pinning of dislocations is weak [13,17]. This leads to a less pronounced manifestation of the stress-drop effect and a more uniform development of plastic deformation at the early stages of loading in EBAM-fabricated steel as compared to the cast one.

To summarize the results, the specimens of the additively manufactured low-carbon steel demonstrate a bit lower values of strength characteristics and ductility in comparison with cast steel within the temperature interval (183–300) K, which covers the climatic temperature range of low-carbon steel utilization. The revealed decrease in the mechanical properties of the EBAM-fabricated steel relative to the traditionally obtained analogs does not reduce the industrial prospects of EBAM technology for the production of machine parts and mechanisms from this type of material. Moreover, postproduction thermal-mechanical treatment can improve the mechanical properties of AM-fabricated material, as was shown for the austenitic steel fabricated by the laser powder bed fusion [22,23].

### 3.3. Temperature Dependence of the Deformation Pattern and Fracture Micromechanisms of Low-Carbon Steel

**Figure 6:**
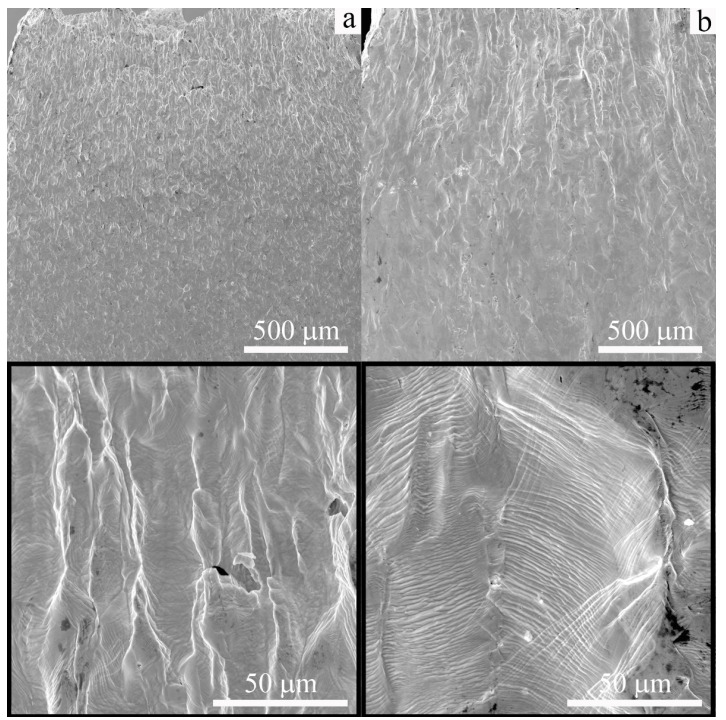
SEM images of the lateral surfaces of the low-carbon steel: (**a**) cast and (**b**) EBAM. Specimens were tensile tested to failure at 300 K.

Figure 6 shows SEM images of the lateral surfaces of the specimens after tensile tests to failure at 300 K. In the case of the cast low-carbon steel, the specimens are deformed uniformly (except the neck region), and the grain of the ferritic phase is elongated along the tensile axis (Figure 6a). In the additively manufactured low-carbon steel deformed at 300 K, deformation traces are also observed, but slip bands and shear bands look finer, and they are more homogeneously distributed than those in cast material (Figure 6b). Single microcracks are seen on the lateral surfaces of the specimens of both types; they open along the slip bands (Figure 6).

**Figure 7:**
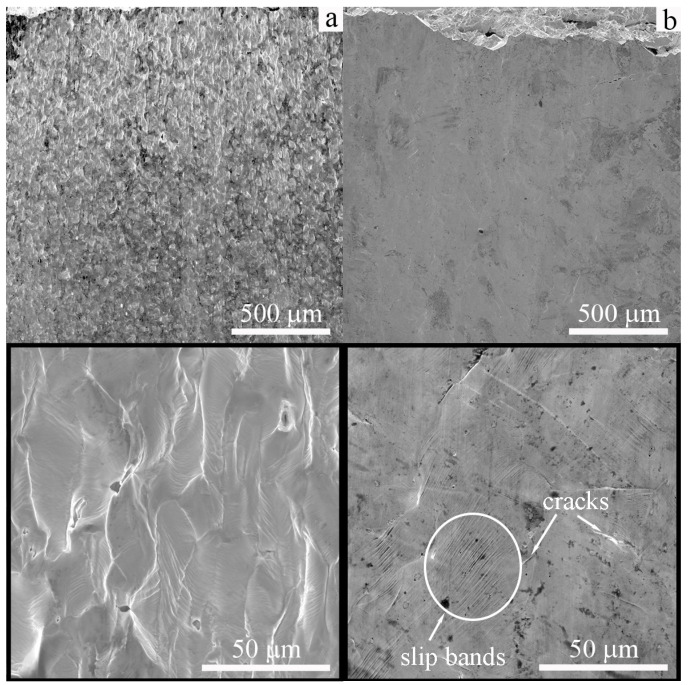
SEM images of the lateral surfaces of the low-carbon steel: (**a**) cast and (**b**) EBAM. Specimens were tensile tested to failure at 77 K.

SEM images of the lateral surfaces of the low-carbon steels subjected to tensile testing at 77 K are shown in Figure 7. Plastic deformation of cast specimens developed by multiple shears (Figure 7a), slip, and twinning is the dominating deformation mechanisms at low temperatures [13]. Microcracks are clearly observed on the lateral surfaces of these specimens. They are not large and located near grain boundaries, macroshear bands, or single large inclusions, which is always presented in conventionally produced steels (Figure 7a). The EBAM-fabricated specimens have smoother side surfaces relative to the cast steel due to the sufficiently lower total elongation (Figure 7b). The magnified image shows very thin shear and slip bands in several slip systems and thin transgranular cracks (Figure 7b).

**Figure 8:**
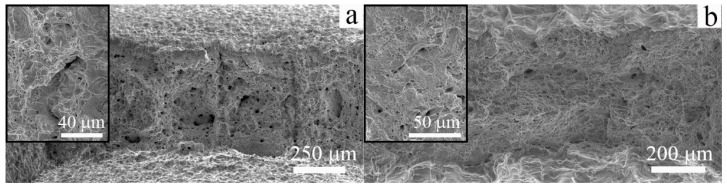
SEM images of fracture surfaces of the low-carbon steel: (**a**) cast and (**b**) EBAM. Specimens were tensile tested to failure at 300 K.

**Figure 9:**
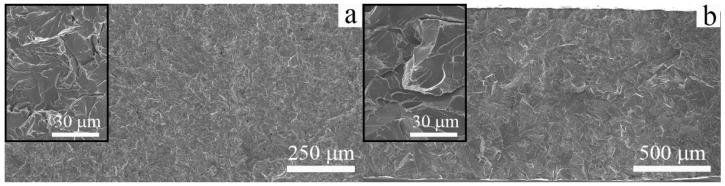
SEM images of fracture surfaces of the low-carbon steel: (**a**) cast and (**b**) EBAM. Specimens were tensile tested to failure at 77 K.

Typical SEM images of the fracture surfaces of the cast and EBAM-fabricated specimens are given in Figure 8. The specimens have been tensile tested to failure at 300 K. Both steels are characterized by a transgranular fracture in the ductile regime. Numerous dimples are observed on the fracture surfaces of the cast specimens (Figure 9a). The specimens of low-carbon steel produced by additive manufacturing have a similar but more uniform dimple morphology of the fracture surfaces; the dimples are coarser (due to the larger grain size) (Figure 8b).

Figure 9 shows SEM images of the fracture surfaces of the cast and the EBAM-fabricated steels after uniaxial tensile tests at 77 K. In both cases, brittle transgranular cracking is observed. It could be seen that, regardless of the method of steel production, the fracture micromechanism is cleavage. The temperature-dependent change in the fracture micromechanism is accompanied by a sharp decrease in the ductility of the additively produced low-carbon steel at 77 K (Table 1), as is similarly and usually observed as a ductile-to-brittle transition in iron and low-carbon steels subjected to impact tests [13]. At the same time, for conventionally produced and normalized low-carbon steel, a change in the fracture micromechanism is not accompanied by a loss of plasticity in the entire temperature range.

Thus, the fracture micromechanisms of low-carbon steel do not depend on the method of its production, but they are a temperature-dependent parameter. A decrease in test temperature from 300 K to 77 K leads to a change in the fracture micromechanism from a ductile to a brittle one. The experimentally observed differences in total elongation of the cast and EBAM-fabricated specimens tested at 77 K arises due to the minor difference in their microstructures. Mechanical twinning is one of the dominating deformation mechanisms of low-carbon steels deformed at 77 K [13]. The flow stress of the steels increases rapidly with decreasing temperature, and the twinning stresses could be reached earlier in the low-temperature deformation regime than at elevated temperatures. The cleavage fracture is strongly assisted by twinning. Cracks nucleate at various twin configurations, such as the intersection of twins in different twin systems or the points where twins interact with grain boundaries [13]. So, the cleavage fracture could be associated with a stress-assisted activation of the mechanical twinning at 77 K in the cast low-carbon steel. The presence of a second phase in the EBAM-fabricated specimens provokes the cleavage fracture as well. In this case, the coarse particles act as additional nucleation sites for cleavages and promote a premature failure of the additively manufactured specimens in a low-temperature deformation regime.

## 4. Conclusions

In the present paper, we investigated the microstructure, phase composition, temperature dependence of the mechanical properties, and mechanisms of deformation and fracture of low-carbon steel produced by the conventional method (normalized) and electron beam additive manufacturing.

Independently of the production method, steel possesses predominantly single-phase ferritic structure with small amounts of perlite (less than 5 vol. %). Additively manufactured low-carbon steel also contains a small number of carbide particles. Therefore, the EBAM-assisted crystallization and multiple heating–cooling cycles of the billet allow for the production of a coarse-grained, mainly ferritic structure in low-carbon steel, which is very similar to that obtained by conventional casting and thermal-mechanical treatments.

Regardless of the production method and testing temperature, plastic deformation of low-carbon steel develops by multiple shears (slip/twinning). The shape of the tensile diagrams for both steels over the entire temperature range is characterized by pronounced yield drops and yield plateaus at the early stages of plastic flow.

Strong temperature dependence of the yield strength is observed. Deforming stresses, the value of the stress drop, and the length of the yield plateau increase with decreasing test temperatures for both steels.

In the temperature interval (183–300) K, the ductility of the specimens almost does not depend on the test temperature. At 77 K, additively produced steel demonstrates a significant decrease in tensile elongation while conventionally fabricated and normalized steel possesses plasticity similar to room temperature deformation.

For both steels, the decrease in test temperature from 300 K to 77 K leads to a change in the fracture micromechanism from dimple fracture to cleavage failure.

Despite the similar phase composition, grain size, deformation behavior, and fracture micromechanisms, the elongation-to-failure of the additively fabricated steel is much lower than that in the conventionally produced counterpart. The presence of small amounts of carbide inclusion, which act as stress concentrators, could assist the nucleation of brittle cracks and cause premature failure of the additively manufactured steel.

## Figures and Tables

**Table 1:** Mechanical properties (yield strength—YS, yield drop value—YD, length of the yield plateau—PL, ultimate tensile strength—UTS, relative elongation—El) of the low-carbon steel in the cast state and after the EBAM process.

Test Temperature, K	The Cast State					After the EBAM Process				
	YS, MPa	YD, MPa	PL, %	UTS, MPa	El, %	YS, MPa	YD, MPa	PL, %	UTS, MPa	El, %
77	701	38	4.4	819	39	662	11	0.8	767	15
183	410	19	3.0	598	40	374	20	1.0	549	34
223	387	17	2.8	554	40	336	19	0.5	510	34
273	371	16	2.6	508	38	298	10	0.5	468	33
300	334	11	2.4	491	38	295	13	0.4	454	33

## Data Availability

Data available on request.
